# Weight bearing or non-weight bearing after surgically fixed ankle fractures, the WOW! Study: study protocol for a randomized controlled trial

**DOI:** 10.1186/s13063-015-0714-1

**Published:** 2015-04-18

**Authors:** Jan Paul Briet, Roderick M Houwert, Diederik PJ Smeeing, Janity S Pawiroredjo, Johannes C Kelder, Koen W Lansink, Luke PH Leenen, Peer van der Zwaal, Stephan WAM van Zutphen, Jochem M Hoogendoorn, Mark van Heijl, Egbert JMM Verleisdonk, Guus W van Lammeren, Michiel J Segers, Falco Hietbrink

**Affiliations:** Department of Surgery, Diakonessenhuis Utrecht, Bosboomstraat 1, Postbus 80250, 3508 TG Utrecht, The Netherlands; Utrecht Traumacenter, Utrecht, The Netherlands; Department of Surgery, University Medical Center Utrecht, Heidelberglaan 100, 3584 CX Utrecht, The Netherlands; Department of Surgery, St. Antonius Hospital Nieuwegein, Postbus 2500, 3430 EM Nieuwegein, The Netherlands; Department of Epidemiology, St. Antonius Hospital Nieuwegein, Postbus 2500, 3430 EM Nieuwegein, The Netherlands; Department of Surgery, St Elisabeth Hospital, Hilvarenbeekseweg 60, 5022 GC Tilburg, The Netherlands; Department of Orthopaedics, Medisch Centrum Haaglanden, Lijnbaan 32, Postbus 432, 2512 VA Den Haag, The Netherlands; Department of Surgery, Twee Steden Ziekenhuis Doctor, Deelenlaan 5, 5042AD Tilburg, The Netherlands; Department of Surgery, Medisch Centrum Haaglanden, Lijnbaan 32, Postbus 432, 2512 VA Den Haag, The Netherlands

**Keywords:** Ankle, Ankle fracture, Lauge Hansen, Post-operative care regimen, Weight bearing

## Abstract

**Background:**

The optimal post-operative care regimen after surgically fixed Lauge Hansen supination exorotation injuries remains to be established. This study compares whether unprotected weight bearing as tolerated is superior to protected weight bearing and unprotected non-weight bearing in terms of functional outcome and safety.

**Methods/Design:**

The WOW! Study is a prospective multicenter clinical trial. Patients between 18 and 65 years of age with a Lauge Hansen supination exorotation type 2, 3 or 4 ankle fractures requiring surgical treatment are eligible for inclusion. An expert panel validates the classification and inclusion eligibility. After surgery, patients are randomized to either the 1) unprotected non-weight-bearing, 2) protected weight-bearing, or 3) unprotected weight-bearing group.

The primary outcome measure is ankle-specific disability measured by the Olerud-Molander ankle score. Secondary outcomes are 1) quality of life (e.g., return to work and resumption of sport), 2) complications, 3) range of motion, 4) calf wasting, and 5) maximum pressure load after 3 months and 1 year.

**Discussion:**

This trial is designed to compare the effectiveness and safety of unprotected weight bearing with two commonly used post-operative treatment regimens after internal fixation of specified, intrinsically stable but displaced ankle fractures. An expert panel has been established to evaluate every potential subject, which ensures that every patient is strictly screened according to the inclusion and exclusion criteria and that there is a clear indication for surgical fixation.

**Trial registration:**

The WOW! Study is registered in the Dutch Trial Register (NTR3727). Date of registration: 28-11-2012.

## Background and rationale

Ankle fractures in adults occur frequently with an annual incidence of approximately 100 to 180 fractures per 100,000 people each year [1-3]. Most ankle fractures occur after inversion or eversion twisting trauma and sports injuries. For stable non-displaced ankle fractures, a conservative treatment with a splint or cast is indicated [4,5]. However, when the congruity of the ankle fork or the joint stability is compromised, open reduction and internal fixation is required to attain full function of the ankle joint [6,7].

While indications for surgical treatment are rather well defined, controversy exists with regard to the optimal postoperative care regimen [8]. Post-operative care regimens vary widely from plaster casts and functional bracing to unprotected non-weight bearing and weight bearing [9-15]. Prior studies show a possible advantage of (protected) weight bearing over (protected) non-weight bearing and of functional mobilization over non-functional mobilization with crutches [11,16]. Some recent, small retrospective and matched control studies even suggested that early (unprotected) weight bearing results in faster recovery and better ankle function [11-13].

The objective of this trial is to compare functional outcome and safety after three different post-operative care regimens: 1) unprotected non weight bearing, 2) protected weight bearing, and 3) unprotected weight bearing.

## Methods and design

### Study design

This is a prospective multicenter randomized controlled trial involving six hospitals in the Netherlands. The study has been approved by the local Institutional Review Board under protocol number WOW-01/NL40835.100.12. This study compares three different post-operative care regimens after ankle surgery; all patients with a Lauge Hansen supination exorotation type 2, 3 or 4 ankle fractures requiring surgical treatment are eligible for inclusion.

**Figure 1:**
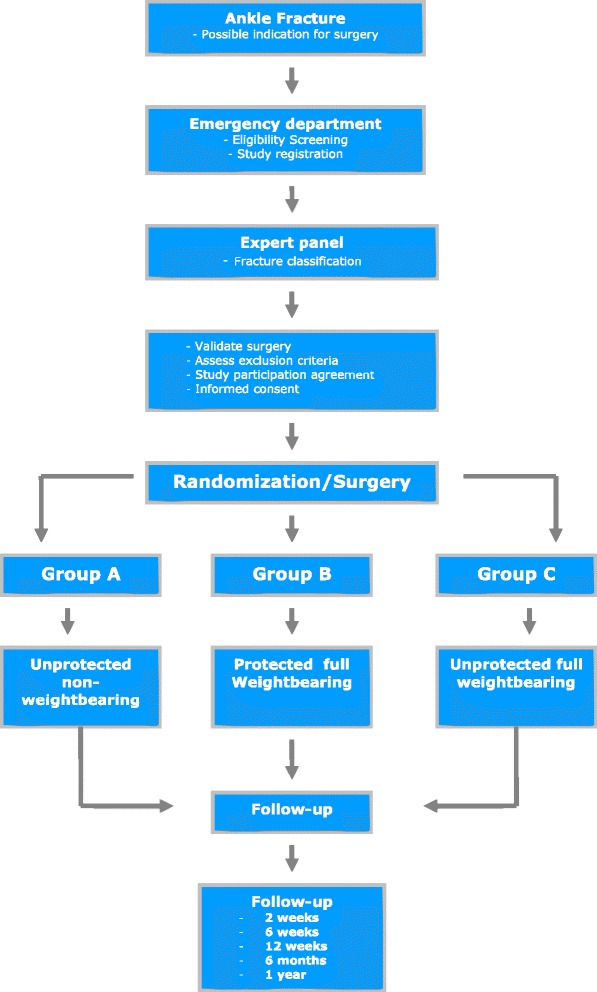
Flowchart WOW! Study.

After registration of the patient, an anonymous X-ray is sent to an expert panel consisting of six experienced orthopedic trauma surgeons. The fracture is classified according to the Lauge Hansen classification and advice on whether or not operative fixation is indicated is provided within 24 hours. A patient is eligible for inclusion when a majority of the expert panel agrees that operative fixation is necessary and the fracture type meets the inclusion criteria. When votes are split equally, the chairman (LL) is the tiebreaker. A flow chart of the study is shown in Figure 1.

### Patient population

Non-pregnant, Dutch speaking patients are being recruited from the participating hospitals in the emergency room or pre-operatively during an outpatient department visit. Patients are screened for eligibility according to the inclusion and exclusion criteria (Table 1). The treating physician or study staff explains the study and informed consent is obtained from each patient. Retrospective data of the participating hospitals shows that approximately 395 patients per year undergo surgery for ankle fractures. Approximately 20% of these patients would be eligible for enrollment according to our inclusion criteria. If this trend continues, we anticipate enrollment of 79 patients per year (Table 1).

**Table 1:** **Inclusion and exclusion criteria**

**Inclusion criteria**	**Exclusion criteria**
Age ranging from 18–65 years	Pre-existent impaired mobility
Fractures classified as Lauge Hansen supination eversion type 2–3 or 4	Expected insufficient stable fracture fixation with standard surgical technique
Articular discongruity of >2 mm on X-ray	Pre-existent cognitive disability
	Necessity for a syndesmosis screw
	Tertius fragment requiring operative fixation
	Body mass index >30
	Diabetes mellitus
	Polytrauma patients (ISS ^a^ >16 or >2 AIS ^b^ regions involved)
	Gustillo 2 and 3 open fractures
	Inability to comply with non-weight-bearing mobilization
	Inability to comply with follow-up

^a^Injury Severity Score.

^b^Abbreviated Injury Scale.

### Inclusion and exclusion criteria

**Figure 2:**
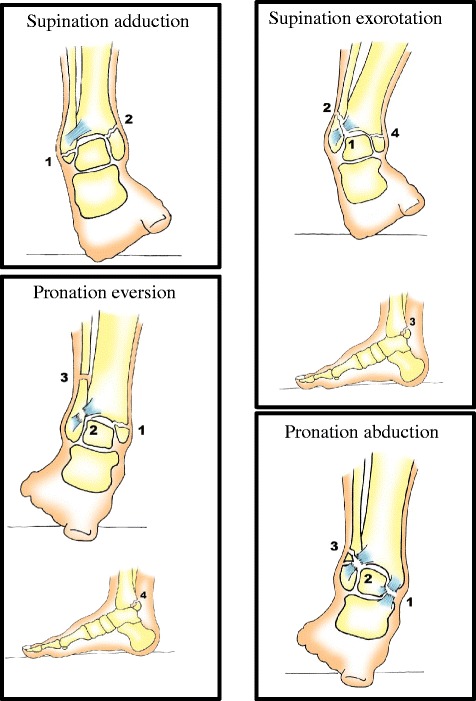
Lauge Hansen classification. Trauma mechanism of ankle fractures. Supination abduction: 1. Talofibular ligament sprain or fibular avulsion; 2. Vertical medial malleolus fracture. Supination eversion: 1. anterior tibiofibular ligament sprain; 2. Lateral oblique fibular fracture; 3. Avulsion of posterior malleolus or ligament rupture; 4. Transverse medial malleolus fracture or disruption of deltoid ligament. Pronation abduction: 1. Transverse medial malleolus fracture or deltoid ligament; 2. Anterior tibiofibular ligament sprain; 3. Transverse comminuted fracture of the fibula. Pronation exorotation: 1. Transverse medial malleolus fracture or deltoid ligament disruption; 2. Anterior tibiofibular ligament disruption; 3. Oblique or spiral fracture of the fibula; 4. Avulsion of posterior malleolus of posterior tibiofibular ligament.

All patients between 18 and 65 years with a Lauge Hansen supination exorotation type 2, 3 and 4 ankle fractures are eligible for inclusion (Figure 2). Exclusion criteria include pre-existent impaired morbidity, cognitive disability, body mass index >30, and diabetes mellitus. All inclusion and exclusion criteria are listed in Table 1.

### Interventions

After enrollment, patients are scheduled for surgery. The ankle fixation technique is ultimately determined by the treating physician; however, advice is provided by the expert panel based on the X-ray. After surgery, all patients are randomized to one of three post-operative care regimens: 1) unprotected non-weight-bearing group – functional weight bearing as tolerated; 2) protected weight-bearing group – weight bearing as tolerated with a below knee cast for 6 weeks; 3) unprotected weight-bearing group – mobilization with crutches, active ankle exercises. All patients see a physiotherapist post-operatively or after cast removal to learn exercises and receive advice on how to start mobilizing according to their specific post-operative care regimen. The physiotherapists have been instructed by the investigators. Patients in the protected weight-bearing group receive low molecular weight heparin as thrombosis prophylaxis for the entire duration of immobilization. For the unprotected non-weight-bearing and unprotected weight-bearing groups, anti-thrombotic treatment is not indicated.

### Randomization process

Patients are randomized using computerized block randomization to either protected weight bearing, unprotected non-weight bearing, or unprotected weight bearing. The randomization is stratified by the participating hospitals. The blocks for randomization consist of 21 patients with the three post-operative care regimens equally represented in each block. Follow-up is by the intention-to-treat principle. Following surgical fixation, the treating surgeon logs into the private website where the patient is randomized to one of three post-operative care regimens.

### Postoperative management and follow-up

Patients are treated during same day admission if possible. According to their post-operative care regimen, patients receive a cast, exercise instructions, and a physiotherapist referral letter. All patients are reviewed in the outpatient clinic by the treating surgeon and/or investigator at 2 weeks, 6 weeks, 3 months, 6 months, and 1 year after surgery. At all post-operative outpatient visits, a standardized clinical exam is conducted including three standardized patient reported outcome questionnaires; the Olerud Molander Ankle Score (OMAS), the Short Form-36 (SF-36), and the Visual Analogue Scale (Table 2).

**Table 2:** **Study assessments by time-point**

**Time point**	**Intake**	**2 weeks**	**6 weeks**	**12 weeks**	**6 months**	**1 year**
OMAS^a^	**X**		**X**	**X**	**X**	**X**
SF-36^b^	**X**			**X**		**X**
Wound inspection		**X**	**X**			
Calf circumference			**X**	**X**	**X**	**X**
Range of motion			**X**	**X**	**X**	**X**
Demographics	**X**					

^a^Olerud Molander Ankle Score.

^b^Short Form 36.

### Primary outcome measures

The OMAS is a scoring scale for symptom evaluation in patients with an ankle fracture and in patients with other acute ankle injuries [17]. This score ranges from 0 to 100%, with 100% representing normal ankle function [17]. It was developed specifically as a comparative research measure to improve consistency and uniformity in ankle injury reporting [17]. A difference of 5 to 10 points between two groups on the OMAS is defined as a clinically relevant result.

### Secondary outcome measures

The SF-36 is one of the most widely used generic health questionnaires. It was developed as a medical outcome score to measure the functional health status of a patient [18,19]. It has been translated into Dutch and validated as a useful questionnaire to assess a broad array of health-related quality-of-life issues [19].

Other secondary measures of function include range of motion (ROM) (plantar and dorso-flexion), weight-bearing pressure load, and calf wasting (difference in calf circumference at enrollment and at 12 weeks post-operative) of the injured ankle. Pain is measured by the Visual Analogue Scale on an 11-point Likert scale. Return to work and sports is also recorded. The pressure load of the injured and non-injured ankle is measured by a scale at every visit, starting at 6 weeks.

Post-operative complications are defined as 1) wound healing problems (no interventions required), 2) superficial wound infections (requiring oral antibiotic treatment based on a wound culture), 3) infection near hardware (requiring surgical debridement and intravenous antibiotic treatment based on a deep tissue culture), 4) hardware failure (requiring re-operation), 5) mal-union or non-union (clinically and radiographically confirmed), and 6) deep venous thrombosis (confirmed by ultrasound).

### Sample size and power

Our hypothesis is that ankle-specific disability assessed with the OMAS is less for unprotected weight bearing when compared to protected weight bearing and unprotected non-weight bearing. An *a priori* power analysis for superiority of treatment with unprotected weight bearing has been conducted for this hypothesis. To detect a clinically significant 7-point difference on the OMAS at 12 weeks follow-up between unprotected non-weight bearing and unprotected weight bearing with a standard deviation of 10, α = 0.05, β = 0.90, two-sided test (based on superiority of unprotected weight bearing), and a maximum loss to follow-up of 20%, a sample size of 75 patients per group is necessary. Therefore, a total of 225 patients are needed for this study.

### Statistical analysis

Analysis will be conducted according to the intention-to-treat principle. In bivariable analysis, Pearson’s correlation will be used for continuous variables, Student’s *t*-test for dichotomous variables such as gender, and ANOVA for categorical variables such as the post-operative care regimen. Multivariate analyses will only be performed *post hoc* and are considered hypothesis-inducing as opposed to hypothesis-testing and will be ascribed as such.

### Data and safety monitoring board (DSMB) and interim analysis

Participation in this trial does not elicit additional risks besides the standard complications of ankle surgery, such as wound infections, deep venous thrombosis, and hardware failure [12,13]. Although all three post-operative care regimens are independently investigated, unprotected weight bearing by unrestricted mobilization as tolerated has not yet been investigated in a randomized trial. Therefore, strict criteria for premature termination are implemented.

A DSMB has been established. An interim analysis will be conducted after every serious adverse event and after half of the target enrollment is reached. The DSMB consists of two independent physicians and one clinical epidemiologist. The members are not committed to this trial. The DSMB will provide advice that will be disclosed to the Institutional Review Board. The steering committee may terminate the study prematurely if advised by the DSMB. In addition, statistically significant, sufficiently powered, and clinically relevant results during interim analysis may be compelling to terminate the trial prematurely. For this trial, the following termination criteria have been established:

- 10% Hardware failure percentage in any of the treatment groups [20,21]
- Wound infection percentage exceeding 20% in any of the treatment groups [14,21]

### Ethical approval

This study will be conducted according to the principles of the Declaration of Helsinki (version 9, October 2008, Seoul) and in accordance with the Medical Research Involving Human Subjects Act. The Verenigde Commissie Mensgebonden Onderzoek (VCMO) approved the study in the St. Antonius Hospital and Diakonessenhuis. The Centrale Commissie Mensgebonden Onderzoek approved the study in Medisch Centrum Haaglanden. The Medisch Ethische Toetsingscommissie approved the study in the St. Elisabeth hospital. Ethical Approval from the Twee Steden hospital was obtained from the board of directors based on VCMO approval.

## Discussion

This trial is designed to compare the effectiveness and safety of unprotected weight bearing with two commonly used post-operative treatment regimens after internal fixation of specified, intrinsically stable but displaced ankle fractures. An expert panel has been established to evaluate every potential subject, which ensures that every patient is strictly screened according to the inclusion and exclusion criteria and that there is a clear indication for surgical fixation.

Prior research reports high patient satisfaction scores and no disadvantage for early weight bearing [12,13,22]. The combination of functional treatment and early weight bearing may reduce soft tissue atrophy and development of osteoporosis and better preserve ankle ROM [13]. Therefore, direct postoperative weight bearing and early mobilization has the potential benefit of earlier functional recovery [8-14,16,23,24].

Ankle fractures in this trial are described by the Lauge Hansen classification. This classification describes the intrinsic stability, demonstrates the trauma mechanism and incorporates ligament injuries [25]. This trial aims to provide evidence for the optimal post-operative care regimen after surgical repair, solely for Lauge Hansen supination exorotation 2–3 and 4 ankle fractures. In contrast to the majority of studies that include patients with all types of ankle fractures requiring operative fixation, this study describes a relatively stable fracture, which may be more suitable for immediate unprotected weight bearing [11-13]. A limitation of the Lauge Hansen classification is its low inter-observer kappa value [26]. The expert panel has been implemented to address this and minimize inter-observer variability.

There are limitations to this study. Not all available post-operative care regimens, such as functional bracing, are studied in this trial, as it has been suggested that functional bracing is associated with an increased risk of post-operative wound healing problems [21].

The termination criteria of this trial exceed the percentages mentioned in literature; the majority of these studies are retrospective studies and small prospective trials [11,20,21]. However, the use of strict termination criteria is important to ensure the safety of the patient.

This prospective randomized controlled trial compares three different post-operative care regimens after open reduction and internal fixation of ankle fractures. By analyzing ankle disability, pain, quality of life, ROM, weight bearing, and resumption of daily activities, this trial assesses the optimal post-operative care regimen for a specific ankle fracture.

## Trial status

The institutional review board has approved the study and patient enrollment has begun in five of the six participating centers. Approval is still pending in one hospital. Recruitment commenced in February 2013 and 73 patients are currently enrolled in this study. Inclusion rates are expected to increase now that most participating centers have received approval. Based on our power analysis and expected yearly inclusion of 79 patients, enrollment of the 225^th^ patient is expected in October 2016. Analysis will be conducted 1 year later once follow-up is completed.

## Abbreviations

DSMB: Data safety monitoring board
OMAS: Olerud Molander ankle score
ROM: Range of motion
SF-36: Short form 36
